# High resolution mapping of traits related to whole-plant transpiration under increasing evaporative demand in wheat

**DOI:** 10.1093/jxb/erw125

**Published:** 2016-03-20

**Authors:** Rémy Schoppach, Julian D Taylor, Elisabeth Majerus, Elodie Claverie, Ute Baumann, Radoslaw Suchecki, Delphine Fleury, Walid Sadok

**Affiliations:** ^1^Earth and Life Institute, Université Catholique de Louvain, Croix du Sud 2, L7.05.14, 1348 Louvain-la-Neuve, Belgium; ^2^School of Agriculture, Food and Wine, Waite Research Institute, University of Adelaide, PMB 1, Glen Osmond, South Australia SA 5064, Australia; ^3^Australian Centre for Plant Functional Genomics (ACPFG), University of Adelaide, PMB 1, Glen Osmond, SA 5064, Australia; ^4^Department of Agronomy and Plant Genetics, University of Minnesota, 411 Borlaug Hall, 1991 Upper Buford Circle, St. Paul, MN 55108, USA

**Keywords:** DREB2A, drought, leaf area, night-time transpiration, phenology genes, Ppd-D1, plant hydraulics, QTL, stomata conductance, *Triticum aestivum*, vapor pressure deficit.

## Abstract

Atmospheric vapor pressure deficit (VPD) is a key component of drought and has a strong influence on yields. Whole-plant transpiration rate (TR) response to increasing VPD has been linked to drought tolerance in wheat, but because of its challenging phenotyping, its genetic basis remains unexplored. Further, the genetic control of other key traits linked to daytime TR such as leaf area, stomata densities and – more recently – nocturnal transpiration remains unknown. Considering the presence of wheat phenology genes that can interfere with drought tolerance, the aim of this investigation was to identify at an enhanced resolution the genetic basis of the above traits while investigating the effects of phenology genes *Ppd-D1* and *Ppd-B1*. Virtually all traits were highly heritable (heritabilities from 0.61 to 0.91) and a total of mostly trait-specific 68 QTL were detected. Six QTL were identified for TR response to VPD, with one QTL (QSLP.ucl-5A) individually explaining 25.4% of the genetic variance. This QTL harbored several genes previously reported to be involved in ABA signaling, interaction with DREB2A and root hydraulics. Surprisingly, nocturnal TR and stomata densities on both leaf sides were characterized by highly specific and robust QTL. In addition, negative correlations were found between TR and leaf area suggesting trade-offs between these traits. Further, *Ppd-D1* had strong but opposite effects on these traits, suggesting an involvement in this trade-off. Overall, these findings revealed novel genetic resources while suggesting a more direct role of phenology genes in enhancing wheat drought tolerance.

## Highlight

Identification of traits and regions in the wheat genome that are involved in night-time and daytime transpiration response to evaporative demand, which can enhance yield-based drought tolerance in wheat.

## Introduction

Drought is the major factor limiting yield production in wheat (Tester and Langridge, 2010). One often-overlooked variable in quantifying drought impact on yield is air vapor pressure deficit (VPD). This variable, which is also termed ‘atmospheric drought’, encapsulates the combined effects of air temperature (T) and relative humidity (RH), and is the main driving force of the whole-plant transpiration rate (TR) (Monteith, 1995). In natural environments, both T and RH contribute to the variation in VPD: on a sunny day, VPD typically increases as T increases and RH decreases progressively throughout the day. In dry environments, this increase takes place during the most part of the day, with VPD values increasing 3–4-fold over a few hours. Because TR and CO_2_ intake both occur through the same leaf anatomical structure, namely the stomata, TR responses to increasing VPD have been linked both theoretically and experimentally to yield under terminal water deficit regimes (Vadez *et al.*, 2014). Recently, Lobell *et al.* (2014) reported that atmospheric VPD has a much stronger effect on current and future yields than previously thought. This effect poses a major challenge for a crop such as wheat whose worldwide yield stagnation has been associated with an increase in the frequency of heat and drought events (e.g. Brisson *et al.*, 2010), both resulting in high-VPD conditions.

The extent of available genetic variability in TR responses to VPD has been investigated in several crops, such as soybean (e.g. Sadok and Sinclair, 2009), maize (Gholipoor *et al.*, 2013), pearl millet (Kholová *et al.*, 2010), and wheat (Schoppach and Sadok, 2012). Despite this progress however, phenotyping and identifying the genetic basis of whole-plant TR response curves to increasing VPD remains undocumented. On pearl millet, Kholová *et al.* (2012) developed a phenotyping approach that allowed for identifying the genetic basis of TR at two different VPD levels, in the morning and the afternoon, but response curves linking TR to increasing VPD were not established. On wheat, several efforts have been undertaken to phenotype wheat transpiration-related traits across populations and environments, but these focused on measuring single leaf conductance (e.g. Condon *et al.*, 1990; Rebetzke *et al.*, 2003, 2013). While revealing a substantial and heritable genetic variability, those efforts were often challenged by leaf-to-leaf variations in stomata conductance and its interaction with VPD fluctuations (e.g. Rebetzke *et al.*, 2013). This further justifies the relevance of attempting an integrative, whole-plant approach to measuring canopy conductance (i.e. the slope of TR response to VPD), while taking into account the strong effect of VPD variations throughout the day. Such effort inevitably requires phenotyping response curves as inputs to a QTL analysis. Because it requires highly accurate whole-plant transpiration and leaf area measurements simultaneously for hundreds of plants under naturally fluctuating conditions, attempting such approach has often been seen as a major phenotyping bottleneck, prompting the need for a new type of high throughput lysimetric phenotyping platforms (e.g. Vadez *et al.*, 2015).

Three groups of other key traits influence whole-plant water use, likely in strong interaction with TR response to VPD. The first is leaf area and related biomass. Like TR, leaf area expansion is strongly influenced by VPD in a genotype-dependent manner (Sadok *et al.*, 2007). However, recent evidence on wheat (Schoppach and Sadok, 2013) and soybean (Devi *et al.*, 2015) suggest that TR and leaf area responses to VPD are coupled, such that TR increases under high VPD are ‘compensated’ by decreases in leaf areas. This indicates that a trade-off exists between both traits that needs to be considered when breeding for either of these traits, making it necessary to investigate their phenotypic and genetic links. Specific leaf area (SLA, the area-to-mass ratio) is a trait that is strongly dependent on leaf area and is often seen as a proxy trait for early vigor (e.g. Rebetzke *et al.*, 2004) and radiation interception and photosynthetic assimilation (e.g. Boote *et al.*, 1996). In wheat, SLA has been shown to have a genetic basis, but it was seldom estimated on a whole plant basis (WPSLA). As a result, the extent of links between SLA, whole-plant leaf area and TR response to VPD remains undocumented. These are important to consider if breeding programs are targeting increasing SLA in drought-prone areas (e.g. Rebetzke *et al.*, 2004).

Another group of traits that are potentially connected to TR response to VPD consists of adaxial and/or abaxial stomata densities (SDAD and SDAB, respectively). Reduction in stomata density has been recently linked to drought tolerance achieved through a decrease in transpiration (Doheny-Adams *et al.*, 2012). So far however no direct links between stomata densities and agronomic yield have been reported in the literature. One possible explanation for that is the lack of evidence of a genetic basis underlying these traits and the absence of a link between stomata densities and whole-plant water use traits such as TR and leaf areas in crop plants. Therefore, phenotyping stomata densities in conjunction with the above traits is likely to provide new answers regarding their genetic control and functional relevance with respect to drought tolerance.

The third is night-time TR (TRN), which until recently was seen to be either marginal or nonexistent, with no clear genetic basis or agronomic relevance with respect to drought tolerance in crops, despite literature pointing to such possibility (Rawson and Clarke, 1988). In wheat, the recent report of Schoppach *et al.* (2014a) indicates that night-time TR can be non-null while being largely controlled by the level of night-time VPD. Further, the study revealed that night-time TR mirrored those observed under daytime conditions, and in particular, the water-saving behavior of a drought tolerant breeding line (RAC875). In the literature, the physiological and/or genetic basis for nocturnal transpiration in plants remains largely unknown with only the investigation of Christman *et al.* (2008) reporting one QTL for nocturnal stomata conductance in Arabidopsis. Investigating the genetic basis of TRN is likely to provide new clues as to its physiological basis and relevance as a drought tolerance trait.

Potentially influencing all of the above, an important feature of wheat physiology is its dependence on major genes that strongly influence phenology by controlling photoperiod sensitivity (*Ppd* genes). In some wheat populations, the effect of these genes is such that their effects are explicitly included as covariates in crop models aiming at predicting key periods for determining yield such as heading time (e.g. Zheng *et al.*, 2013). However, there is evidence that even among populations flowering within small time windows, the effects of genes such as *Ppd-D1* and *Ppd-B1* on yield related-traits under drought are large (Bonneau *et al.*, 2013), suggesting direct effects of those genes on drought tolerance traits. Examining the effects of such maturity genes on each of the considered traits will shed new lights on the ways development and functional traits interact to achieve drought tolerance.

The objective of this study was to examine the genetic basis of the above traits and their dependence on phenology genes within a wheat mapping population consisting of 143 double haploid (DH) lines descending from a cross between a conservative, drought-tolerant donor parent (RAC875) and a check cultivar (Kukri) at a genetic resolution that readily offers access to genomic and molecular information. To this end, we developed a methodological framework combining (i) a new, high throughput phenotyping method (ii) a high-density genetic map (iii) a whole-genome QTL analysis and (iv) a genomic approach for identifying putative drought-tolerance genes.

## Materials and methods

### Genetic material

The genetic material used in the study consisted of a set of bread wheat (*Triticum aestivum* L.) 143 DH lines descending from the F1 generation of a cross between the drought tolerant donor line RAC875 (RAC655/3/ Sr21/4*Lance//4*Bayonet) and the check cultivar Kukri (76ECN44/76ECN36//Madden/6*RAC177). RAC875 has been reported to display a conservative TR in response to soil water deficit in comparison to Kukri (Schoppach and Sadok, 2012). Both genotypes were developed by the Roseworthy Agricultural Campus and the University of Adelaide. The 143 DH lines were randomly selected among a group of lines that flowered within a narrow window of ~3d.

### Sowing and growth conditions

The plants were sown on 7 May 2014 and grown for 36 d in a new automated glasshouse at the Université catholique de Louvain, Belgium (50°40ʹN, 4°36ʹE). During the growth period, the glasshouse was programmed for a daytime T setting of 20 °C (min.) and 35 °C (max.), resulting in average daytime/night-time T and VPD values of 28.6 °C (±0.5 SE) and 2.25 kPa (±0.10 SE)/22.8 °C (±0.3 SE) and 1.3 kPa (±0.03 SE) respectively. The glasshouse system provided a supplementary lighting that was activated automatically each time incident daytime photosynthetic photon flux density (PPFD) levels dropped below 500 μmol m ^−2^ s^−1^ by means of a high intensity photosynthetic LED lighting system (~200 μmol m^−2^ s^−1^ at canopy level, Pro 650, LumiGrow, Novato, California, USA).

Three replicate plants per genotype including both parents were sown at a depth of 2.5cm in 435 custom-made PVC columns (0.11 m diameter and 0.33 m tall), each filled with 1.40–1.45kg of compost garden soil (DCM Corporation, Grobbendonk, Belgium). The pots were covered with aluminum foil to avoid excessive soil heating and six small holes were drilled in the bottom of the pots to allow for drainage. Prior to sowing, all the seeds were weighted and only those with weights in the 0.030–0.035g range were selected. Seven days after sowing, each pot was thinned to a single plant. Pots were watered every 2–3 d during the first 2 weeks and then daily until the experiment was initiated, 36 d after sowing. During the growth period, in addition to the glasshouse sensors, T and RH conditions were continuously recorded in the immediate vicinity of the plants every 5min by shielded pocket sensors connected to USB dataloggers (EL-USB-2-LCD, Lascar Electronics, Whiteparish, UK) placed in five locations across the setup.

### Phenotyping transpirational water loss

Daytime TR responses to VPD were carried out as in Schoppach and Sadok (2012), that is, under naturally fluctuating greenhouse conditions, where VPD increased progressively throughout a large portion of the day. On the day before the TR measurements began, the pots were watered to dripping around 17:00 solar time and aluminum foils were immediately placed over the soil and sealed around the stem of the plant to eliminate direct soil evaporation. The pots were then allowed to drain overnight. The following morning, the measurements were initiated on the fully well-watered plants by weighting the pots every hour from 06:00 to 13:00 solar time. During this time period, the incident PPFD exceeded the saturating value of 503 μmol m^−2^ s^−1^ starting from the second TR measurement until the end of the experiment.

Five persons performed the weightings simultaneously by placing the pots on five identical balances with a resolution of 0.01g (Model Fx-3000i, A & D Co. Ltd, Tokyo, Japan) connected to five dataloggers, thereby making it possible to perform hourly weightings within 25–30min. During this time period, the average hourly air VPD inside the glasshouse continuously increased from 1.1 to 3.7 kPa over a period of 7h (instantaneous RH decreasing from 60.2 to 33.3% and T increasing from 20.9 to 38.5 °C). During the weightings, T and RH measurements were recorded every 5min in nine locations across the setup by the same pocket sensors as those mentioned previously. For each genotype, the transpirational water loss at a given VPD was calculated as the difference in pot weight (normalized by leaf area) over a period of 60min while averaging the VPD values during the same period. Repeating the procedure over the course of the 7h ensured that regressions characterizing the slopes (SLP) of whole-plant TR response curves to increasing VPD could be constructed (Sadok and Sinclair, 2009; Schoppach and Sadok, 2012). At the end of the weighting sequence, the plants were re-watered to dipping. All plants were weighted later that day at ~23:00 and the following morning at around 04:00, over a period during which night-time VPD was constant at 1.3 kPa and PPFD at 0 μmol m^−2^ s^−1^. Night-time TR (TRN) was estimated for each genotype as the difference in pot weight over the 5h period, normalized by plant size (i.e. leaf area). The daytime phenotyping approach was replicated during an independent experiment carried out on 26 April 2015 on eight select genotypes displaying SLP values that covered the entire phenotypic range of the population. The plants were grown for 37 d in the same environment as described above and whole-plant TR was determined in the same way, except that it was measured at a constant VPD of 2.5 kPa in a walk-in growth chamber, where PPFD was kept constant at ~450 μmol m^−2^ s^−1^. These TR values were examined in relation to those measured during the 2014 phenotyping experiment at the same VPD of 2.5 kPa, and under higher PPFD (850 μmol m^−2^ s^−1^). This allowed for testing the repeatability of the TR measurements over different durations and light environments.

### Phenotyping whole-plant leaf area and biomass

After measuring transpirational water loss, whole-plant leaf areas (WPLAs) were measured using a high-precision leaf area meter (LI- 3100C, Li-Cor, Lincoln, NE, USA) connected to a laptop. As indicated above, the resulting values were used for normalizing TR to account for differences in plant size and also recorded as independent phenotypic variables. Afterwards, whole-plant leaf dry weights, tiller and spike dry weights (TSW), Zadoks growth scale (ZS) score and number of tillers (NTIL) were recorded. The dry weights were measured using a precision balance with a resolution of 0.001g after placing the samples inside a drying oven at 60 °C for 7–8 d. Whole-plant leaf dry weights and WPLA were used to calculate the whole-plant specific leaf area (WPSLA), the ratio of leaf area to its dry weight (cm^2^ g^−1^).

### Phenotyping stomata densities

Given the high number of plants, a simple and new method for rapidly measuring stomata densities was devised. During leaf area measurements, 3 cm-long leaf segments were cut in the mature region of the top, most fully developed leaf of each plant. The segments were then fixed in a FAA solution (ethanol, formaldehyde, acetic acid) for 1 week and then transferred to a bleaching solution (ethanol, acetic acid) for 3 d before they were stored in a 70% ethanol solution. Stomata densities were then determined for the abaxial and adaxial sides using a light microscope (Eclipse E400, Nikon Corporation Instruments Company, Japan) equipped with a digital camera. On each side, the adaxial (SDAD) and abaxial (SDAB) stomata densities were determined on the basis of three major vein-free areas each covering 0.915mm^2^ (magnification ×200).

### Linkage map

The genetic linkage map used for analysis in this research was the new integrated SSR-DArTs-SNP linkage map described in Mahjourimajd (2015). The linkage map was constructed using the package ASMap (Taylor and Butler, 2014) available in the R statistical computing environment (R Development Core Team, 2015) and contains 15911 markers genotyped on 218 individuals. The marker set consisted of 408 SSR and DArT markers from the original linkage map described in Bennett *et al.* (2012) and 15503 SNP markers from an Illumina 90K SNP array. The linkage map spanned 26 linkage groups with 1333 unique loci, a total length of 2864.3 cM and an average inter-marker distance of 2.18 cM. This represented a dramatic reduction from the 7.32 cM average inter-marker distance in the SSR-DArTs linkage map of Bennett *et al.* (2012) and implied significantly higher resolution QTL mapping is achievable with the integrated SSR-DArTs-SNP linkage map. To ready the map for analysis the alleles for the 1333 unique markers were given numeric values (AA=1; BB=−1) and missing alleles were imputed using the flanking marker rules of Martinez and Curnow (1992). Pseudo-interval markers were then calculated at the mid-point between markers using the method derived in Verbyla *et al.* (2007).

### Linear mixed model analysis

After an exploratory analysis of the traits, it was found ZS exhibited significant non-normality and was excluded from further analysis. For the remaining traits, an initial analysis was conducted using a linear mixed model which accounted for genetic sources of variation from the DH lines as well as non-genetic sources of variation arising from the design of the experiment. The fixed component of the linear mixed model contained a factor consisting of a level for all the DH lines and one for each of the parents, RAC875 and Kukri. The inclusion of this term ensured the parents remained fixed in the analysis. Given that this population was known to segregate for phenology loci *Ppd-B1* and *Ppd-D1*, the effect of these genes had to be accounted for as in Bonneau *et al.* (2013). To ensure the genetic component of the model was appropriately adjusted for phenology loci, maturity genes *Ppd-B1* and *Ppd-D1,* numerically coded as AA=1 and BB=−1 with missing values set to zero to reflect the uncertainty of the allele call, were added to the fixed component of the model as covariates (Bonneau *et al.*, 2013). Extraneous non-genetic variation, such as replicate effects, were captured using random effects. The random component of the model also contained a genetic term consisting of a factor with a level for each of the DH genotypes and parents. As the parents were fixed, the genetic variance for this term was associated with the DH lines only. With the exception of SLP, appropriate information was extracted from each of the fitted trait models and broad sense heritabilities were calculated using the formula derived in Cullis *et al.* (2006). To provide an indication of the heritable nature of TR across the measured VPD levels, additional linear mixed models were fitted for whole-plant TR at the lowest (1.1 kPa, TR1.1) and highest VPD (3.7 kPa, TR3.7) levels (Kholová *et al.*, 2012). The information from these models was then used to calculate broad sense heritabilities for these two additional traits.

### Multi-trait linear mixed model analysis

To provide an accurate assessment of the genetic inter-relatedness of the nine traits a multi-trait linear mixed model analysis was conducted. Preceding analysis, each of the traits was standardized by subtracting its mean and dividing through by its standard deviation. The fixed component of the multi-trait model contained trait by *Ppd-D1* and trait by *Ppd-B1* terms to model the phenology genes for each trait. Non-genetic sources of variation such as replicate effects were modeled using a separate variance for each trait. An important aspect of the multi-trait linear mixed model was the addition of terms that accurately model the relatedness of the traits at the residual and genetic level. Consequently, the variance of the residuals was assumed to have an unstructured variance-covariance matrix with residual variances for each of the traits on the diagonal and covariances on the off diagonals to capture the connection between traits at the residual level. Similarly, an unstructured variance-covariance structure was assumed for the variance of the genotype by trait interaction random effect term. The diagonal components of this matrix consisted of genetic variances of the DH lines for each of the traits and the off diagonals were genetic covariances that reflected the relationships of the DH lines between traits. From the fitted multi-trait model the estimated genetic variance-covariance structure was extracted and a genetic correlation was calculated. All univariate and multi-trait linear mixed model analyses were performed using the flexible linear mixed modeling package ASReml-R (Butler *et al.*, 2009) available in the R statistical computing environment.

### QTL analysis

For each trait a QTL analysis was undertaken using the whole genome average interval mapping (WGAIM) approach of Verbyla *et al.* (2007, 2012). This approach uses a whole genome procedure for detection and estimation of QTL through extensions of the established linear mixed model for each trait. Initially, a working linear mixed is derived that incorporates the whole genome pseudo-interval marker set as a contiguous block of random effect covariates with zero mean and a single marker variance parameter. This variance parameter is then tested and if found significant an outlier statistic profile is used to identify and select the most likely interval containing the putative QTL. This interval is then removed from the contiguous block and placed as a separate random effect. This process is repeated until the marker variance parameter is not significant and no more putative QTL are detected. The combination of this forward selection approach and the implementation of the high dimensional methodology outlined in Verbyla *et al.* (2012) ensures WGAIM is efficient for small to moderate population sizes with large sets of genetic markers. The WGAIM approach is computationally implemented in the R package wgaim (Taylor and Verbyla, 2011) available in the R statistical computing environment. The package provides detailed summaries of significantly detected QTL including their position, effect size, LOD score and contribution to the genetic variance. Additionally, it contains a function to plot the linkage map with significant QTL intervals highlighted for multiple traits simultaneously.

### Gene identification and RNA-Seq data

SNPs underlying the target QTL were assigned to contigs of the wheat chromosome survey sequences (CSS) based on the information provided by Wang *et al.* (2014). To obtain gene expression information for genes located in the QTL region we used the PopSeq map for bread wheat together with the high confidence (HC) gene predictions described in IWGSC (2014) and the publicly available RNA-Seq data for cv. Chinese Spring (http://wheat-urgi.versailles.inra.fr/Seq-Repository/RNA-Seq). This dataset covers five different organs (root, leaf, stem, spike, grain) at three developmental stages, each in two replicates. We used the HC gene predictions, version 2.1 (ftp://ftpmips.helmholtz-muenchen.de/plants/wheat/IWGSC/genePrediction_v2.1/) as reference for mapping the RNA-Seq reads. The reference was prepared by extracting the genomic sequence for each of the predicted genes with up to 2kb upstream and downstream bases whenever available from the corresponding IWGSC-CSS contig. The coordinates in the corresponding GTF/GFF transcript annotations file were adjusted accordingly. RNA-Seq reads were quality-, adapter- and length-trimmed using Trimmomatic (Bolger *et al.*, 2014), version 0.30, with a custom list of adapter sequences and the following settings: ‘ILLUMINACLIP:adapters.fa:1:6:6 LEADING:3 TRAILING:3 SLIDINGWINDOW:4:6 MINLEN:60’. After indexing the reference using Bowtie2 (Langmead and Salzberg, 2012) version 2.2.1, trimmed reads were aligned to the reference using TopHat (Kim *et al.*, 2013) version 2.2.1, not allowing any mismatches or indels. Paired reads were required to map concordantly (--no-discordant setting) to the same (--no-mixed setting) reference sequence. BAM files for the biological replicates were merged, apart from a single tissue/stage sample (spike_Z39) where only one sample was available. Expression was quantified by Cufflinks (Roberts *et al.*, 2011) version 2.1.1 utilizing (through the --GTF option) the adjusted version of the reference transcript annotations provided with the HC gene predictions. The remaining settings were left at their defaults, except for: --max-multiread-fraction 1, --frag-len-std-dev 50 --max-intron-length 5 000. FPKMs (fragments per kilobase of exon per million fragments mapped) per gene (rather than per isoform) were extracted and aggregated in tabular form. Functional annotation was obtained by comparison of the HC gene predictions to MSU Rice Genome, Release 7, and Arabidopsis TAIR using BlastX (expect-value <10^−10^).

## Results

### Variability of whole-plant daytime TR response curves and night-time transpiration

**Fig. 1.**
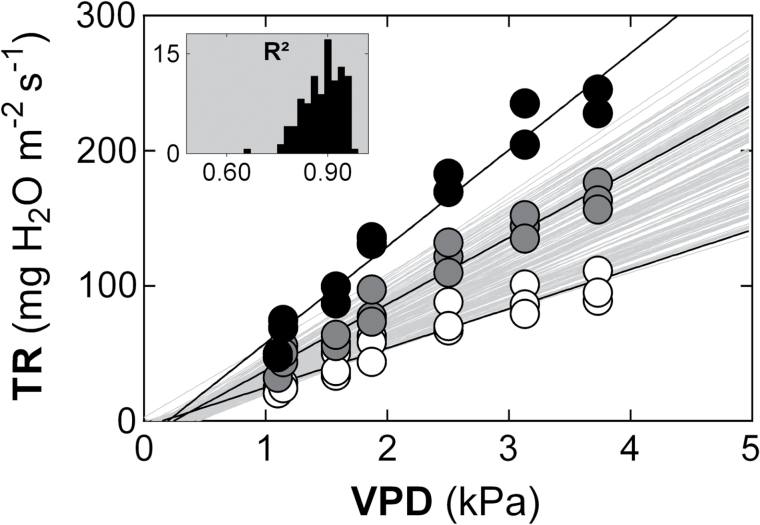
Genetic variability of whole-plant TR responses to increasing VPD within the mapping population of 143 DH lines from the RAC875×Kukri cross. The regressions and the datapoints of three typical genotypes are highlighted for genotypes DH_R127 (highest slope, black circle), DH_R081 (average slope, gray circle) and DH_R129 (lowest slope, open circle), with the background light gray regressions representing those of the remaining 140 lines. The values of the slopes (mg H_2_O m^−2^ s^−1^ kPa^−1^) of these genotypes are 71.5 (R^2^=0.96), 49.2 (R^2^=0.96) and 29.1 (R^2^=0.91), respectively. The black histograms of the insert represent the distribution of the R^2^ values for all the regressions.

**Fig. 2.**
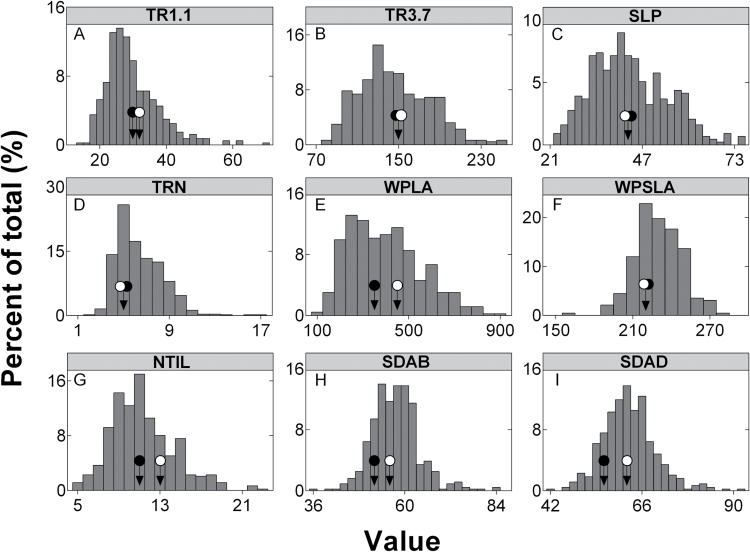
Frequency distributions of the traits measured for the RAC875×Kukri DH population of 143 lines. Black and white circles indicate the positions of the parents RAC875 and Kukri, respectively. The units for the traits are: mg H_2_O m^−2^ s^−1^ (TR1.1, TR3.7, TRN); mg H_2_O m^−2^ s^−1^ kPa^−1^ (SLP); cm^2^ (WPLA); cm^2^ g^−1^ (SLA); mm^−2^ (SDAB, SDAD).

For each genotype a total of 21 daytime whole-plant TR measurements (seven replicated three times) were carried out in the 1.1 to 3.7 kPa range simultaneously for the entire population (Fig. 1). The corresponding lowest (TR1.1) and highest (TR3.7) TR values were significantly variable between genotypes, reflecting a highly transgressive segregation in this population (Fig. 2A, B). The genetic variability of TR3.7 was large, ranging between 94.1 and 236.7mg H_2_O m^−2^ s^−1^, dependent on the genotype. This variability was confirmed for TR values measured at the lowest VPD (TR1.1), which ranged from 20.3 to 55.1mg H_2_O m^−2^ s^−1^ kPa^−1^. This variation had a strong genetic basis as indicated by the broad sense heritabilities of 0.7 and 0.61 for TR1.1 and TR3.7, respectively.

Characterizing the response of whole-plant TR to increasing daytime VPD for each genotype of the population required a total of 145 regressions analyses (Fig. 1). A departure from linearity analysis returned non-significant effects for all of the genotypes. It was therefore considered that a linear model was the best descriptor of TR responses to VPD for this population. Overall, the observed linear regressions were of a good quality, returning R^2^ values ranging from 0.77 to 0.97 (average value: 0.88, Fig. 1) with the exception of one extreme R^2^ outlier. There was a strong transgressive segregation for the resulting slopes (SLP, Fig. 2C), associated with a large variability, which ranged from 27.6 to more than 2.5 times this value, at around 71.6mg H_2_O m^−2^ s^−1^ kPa^−1^. This variability was confirmed in the independent validation experiment carried out on eight genotypes (DH_R003, 035, 054, 070, 109, 112, 127, 129) that covered the entire range of SLP values, revealing TR values that were highly correlated with those previously measured (r=0.97; *P*<0.0001).

Similarly, whole-plant TRN varied greatly among the genotypes, ranging from 3.5 to 13.1mg H_2_O m^−2^ s^−1^. This variability was under a strong genetic control, as indicated by the observed broad sense heritability for TRN (0.73). Similar to SLP, the distribution of TRN revealed a strong transgressive segregation (Fig. 2D).

### Variability of whole-plant evaporative surface, specific leaf area, stomata densities and number of tillers

Whole-plant leaf area (WPLA) and specific leaf area (WPSLA), abaxial and adaxial (SDAB and SDAD) stomata densities and the number of tillers per plant (NTIL) varied substantially among the genotypes. This variation had a strong genetic basis as indicated by the high heritabilities for these traits, which were 0.91, 0.71, 0.69, 0.63 and 0.67, respectively. Further, as indicated in Fig. 2, a transgressive segregation was observed for all these traits. All those traits, with the exception of ZS (which ranged from 31 to 61) were normally distributed.

### Effect of the *Ppd-D1* and *Ppd-B1* genes

**Fig. 3.**
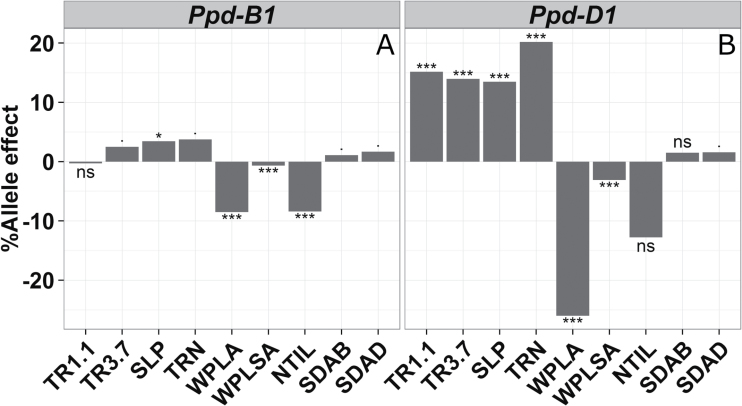
Allele effects of the maturity genes *Ppd-B1* (A) and *Ppd-D1* (B) presented as the average percentage change in the trait mean when the genotype is carrying the AA (RAC875) allele. The symbols ., *, *** denote significant differences at the 0.05, 0.01 and <0.001 probability levels respectively. ns, non-significant effect.

The effects of the phenology genes *Ppd-D1* and *Ppd-B1* on the measured traits were significant but substantially different (Fig. 3). For both genes, the presence of the RAC875 allele resulted in an increase of the value of trait means of those directly involved in transpirational water loss (TR1.1, TR3.7, SLP, TRN, SDAB and SDAD) while resulting in a decrease in the values of WPLA, WPSLA and NTIL. Overall, the alleles of *Ppd-D1* had much stronger effects compared to those of *Ppd-B1*, with the exception of stomata density traits (SDAB and SDAD), which were weakly influenced by both genes.

### Phenotypic and genetic correlations

Expectedly, whole-plant daytime transpiration traits TR1.1, TR3.7, SLP were highly and positively correlated both phenotypically and genotypically (Table 1). In the same way, those traits positively and strongly correlated with TRN. All transpiration traits correlated negatively (phenotypically and genotypically) with WPLA, WPSLA and NTIL in a decreasing order in terms of strength. In strong contrast to the above, stomata density traits correlated weakly with all traits, while exhibiting strong inter-correlations (Table 1). Stomata density traits were systematically positively correlated with daytime and night-time TR.

**Table 1.** Phenotypic (upper half) and genotypic (lower half) correlations among the studied traits

**Traits** ^**a**^									
	**TR1.1**	**TR3.7**	**SLP**	**TRN**	**WPLA**	**WPSLA**	**NTIL**	**SDAB**	**SDAD**
**TR1.1**		0.74	0.64	0.68	−0.69	−0.54	−0.47	0.18	0.19
**TR3.7**	0.96		0.97	0.75	−0.77	−0.64	−0.49	0.24	0.23
**SLP**	0.95	0.99		0.75	−0.72	−0.63	−0.45	0.24	0.22
**TRN**	0.89	0.91	0.90		−0.75	−0.57	−0.51	0.22	0.20
**WPLA**	−0.82	−0.91	−0.93	−0.76		0.63	0.73	−0.25	−0.24
**WPSLA**	−0.69	−0.76	−0.77	−0.65	0.77		0.35	−0.34	−0.32
**NTIL**	−0.60	−0.67	−0.71	−0.54	−0.81	0.58		−0.11	−0.11
**SDAB**	0.34	0.41	0.42	0.32	−0.42	−0.53	−0.21		0.90
**SDAD**	0.22	0.30	0.32	0.21	−0.37	−0.48	−0.19	−0.99	

^a^ See abbreviations for details. All phenotypic correlations were significant at the 0.05 to 0.0001 levels.

### QTL detection

**Fig. 4.**
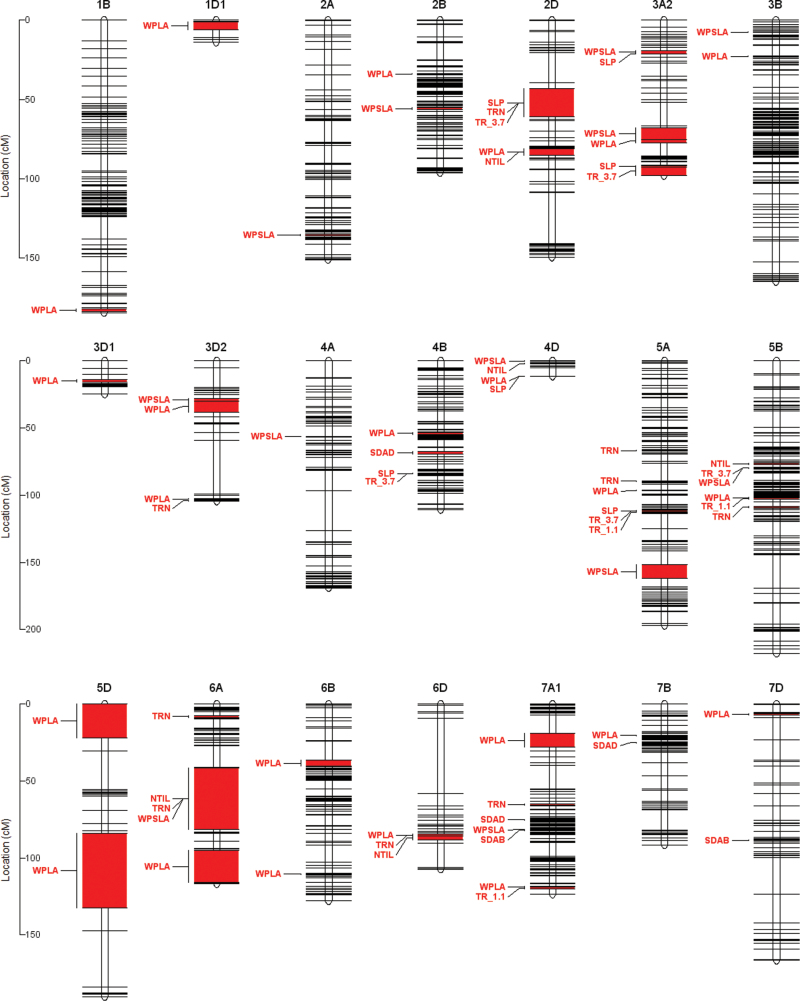
QTL location on the RAC875/Kukri linkage map. The genetic distances (cM) are reported on the scales to the left of the chromosomes. Each horizontal black line represents one of the unique 1333 marker positions on the genome (for information about the traits, see abbreviations). (This figure is available in color at *JXB* online.)

In total, 68 novel, mostly trait-specific QTL were detected across 21 linkage groups (Fig. 4). Further, 47% of these QTL had peaks that were located in a narrower region of 0.17–0.46 cM. No QTL were detected for TSW.

A total of eight QTL were detected for TR1.1 and TR3.7 (Table 2). All of the eight QTL, with the exception of a relatively minor one detected for TR3.7 on chromosome 5B, were specific to daytime TR. One QTL, detected on chromosome 5A was common for both TR1.1 and TR3.7 explaining 19.3% and 21.6% of the genetic variance, respectively (Table 2). Additionally, TR1.1 and TR3.7 were each controlled by distinct major QTL on chromosomes 5B and 2D explaining 11.1% and 20.8% of the genetic variance, respectively (Table 2). Overall, the RAC875 allele decreased transpiration for six out of the eight QTL detected.

**Table 2.** Significant QTL detected for the studied traits (major QTL shown in bold)

**Trait** ^**a**^	**QTL name** ^**b**^	**Left marker** ^**c**^	**Position (cM**)^**d**^	**Right marker** ^**e**^	**Effect** ^**f**^	**%Var** ^**g**^	**LOD** ^**h**^
TR1.1	**QTR1.1.ucl-5A**	**BobWhite_c40643_370(C**)	**112.37–112.82**	**Tdurum_contig52695_323(C**)	**−2.08**	**19.3**	**5.5**
	**QTR1.1.ucl-5B**	**BobWhite_c47456_121(C**)	**102.04–102.96**	**BS00009335_51(C**)	**−1.54**	**11.1**	**3.1**
	QTR1.1.ucl-7A	Excalibur_c1791_819(C)	118.95–119.87	BobWhite_c19346_434(C)	1.40	9.4	2.8
TR3.7	**QTR3.7.ucl-5A**	**Ex_c27046_1546**	**111.45–112.37**	**BobWhite_c40643_370(C**)	**−8.65**	**21.6**	**7.3**
	**QTR3.7.ucl-2D**	**wsnp_CAP12_ c1503_764765**	**43.18–60.86**	**Ex_c10377_845(C**)	**−9.26**	**20.8**	**3.0**
	**QTR3.7.ucl-3A**	**IAAV902(C**)	**92.39–97.94**	**wsnp_Ex_c26887_36107413(C**)	**−5.51**	**8.7**	**3.3**
	QTR3.7.ucl-5B	BobWhite_c8048_663(C)	79.57–80.03	BobWhite_c45340_368(C)	−5.14	8.2	2.6
	QTR3.7.ucl-4B	BobWhite_c4818_173(C)	83.61–84.07	Excalibur_c36457_100(C)	4.78	7.2	2.5
SLP	**QSLP.ucl-5A**	**Ex_c27046_1546**	**111.45–112.37**	**BobWhite_c40643_370(C**)	**−2.44**	**25.4**	**6.7**
	**QSLP.ucl-2D**	**wsnp_CAP12_ c1503_764765**	**43.18–60.86**	**Ex_c10377_845(C**)	**−2.48**	**22.3**	**2.4**
	**QSLP.ucl-4B**	**BobWhite_c4818_173(C**)	**83.61–84.07**	**Excalibur_c36457_100(C**)	**1.60**	**11.6**	**3.1**
	**QSLP.ucl-3A.2**	**BS00064039_51(C**)	**91.93–92.39**	**IAAV902(C**)	**−1.50**	**10.3**	**3.0**
	QSLP.ucl-4D	wsnp_Ex_ c34252_42593715	11.37–11.54	IAAV5607	1.27	7.3	2.1
	QSLP.ucl-3A.1	wsnp_Ex_ c44375_50444862	21.12–21.35	BobWhite_c11935_137(C)	1.16	6.5	1.9
TRN	**QTRN.ucl-5A.2**	**BobWhite_c14291_385(C**)	**89.37–89.83**	**BobWhite_rep_c63943_76(C**)	**−0.49**	**11.7**	**4.1**
	**QTRN.ucl-2D**	**wsnp_CAP12_ c1503_764765**	**43.18–60.86**	**Ex_c10377_845(C**)	**−0.54**	**11.5**	**2.8**
	**QTRN.ucl-5B**	**IACX2901(C**)	**108.47–109.38**	**BobWhite_c48435_165(C**)	**−0.44**	**9.5**	**4.5**
	**QTRN.ucl-6A.1**	**Excalibur_c4483_1053(C**)	**7.33–8.7**	**wsnp_Ex_c431_848310(C**)	**−0.41**	**8.2**	**4.2**
	QTRN.ucl-6A.2	wsnp_Ex_ c2389_4479352(C)	41.61–81.29	barc0353b(C)	0.50	7.9	3.7
	QTRN.ucl-5A.1	BS00063735_51(C)	66.58–67.04	Excalibur_c54514_248(C)	0.36	6.5	2.2
	QTRN.ucl-3D	barc0284(C)	103.99–104.45	wsnp_Ku_c7264_12545135(C)	0.36	6.4	4.0
	QTRN.ucl-6D	Kukri_c31995_1948(C)	84.37–85.82	wsnp_Ra_c13881_21836489	0.36	6.3	3.8
	QTRN.ucl-7A	BS00063267_51	64.64–65.55	IACX9283(C)	0.25	3.1	1.8
SDAB	**QSDAB.ucl-7A**	**BS00065822_51(C**)	**81.77–82.23**	**wPt.8399(C**)	**1.56**	**15**	**3.6**
	QSDAB.ucl-7D	CAP8_rep_c9420_186(C)	88.21–88.66	D_GBB4FNX01CLBM2_184(C)	1.27	10.3	2.6
SDAD	**QSDAD.ucl-7A**	**Kukri_c29386_182(C**)	**74.92–75.38**	**BS00067200_51(C**)	**2.03**	**23.6**	**6.2**
	**QSDAD.ucl-4B**	**tplb0061a20_153(C**)	**67.44–69.38**	**wsnp_Ex_c5187_9195120(C**)	**1.57**	**14.1**	**3.8**
	QSDAD.ucl-7B	wsnp_Ku_ c11060_18147688(C)	24.78–25.24	wsnp_Ra_c31052_40235870(C)	1.18	8.4	2.5
NTIL	**QNTIL.ucl-2D**	**RAC875_c39665_175(C**)	**81.1–85.23**	**Ex_c2115_3369(C**)	**0.75**	**18.4**	**5.5**
	**QNTIL.ucl-5B**	**Excalibur_c1892_1521**	**76.36–77.28**	**Excalibur_c40672_657(C**)	**0.56**	**11.5**	**3.2**
	**QNTIL.ucl-4D**	**wsnp_Ku_rep_ c109720_94223856(C**)	**1.82–2.28**	**wsnp_Ex_rep_ c79748_75305162(C**)	**−0.54**	**10.1**	**3.2**
	QNTIL.ucl-6A	wsnp_Ex_ c2389_4479352(C)	41.61–81.29	barc0353b(C)	−0.64	9.5	2.8
	QNTIL.ucl-6D	wsnp_Ra_ c13881_21836489	85.82–88.39	barc0096(C)	−0.43	6.8	2.3
WPLA	**QWPLA.ucl-5B**	**Tdurum_ contig12551_233(C**)	**101.58–102.04**	**BobWhite_c47456_121(C**)	**45.12**	**15.8**	**18.9**
	**QWPLA.ucl-2B**	**Excalibur_c40056_59(C**)	**33.72–34.18**	**barc0007(C**)	**−33.29**	**9**	**6.2**
	**QWPLA.ucl-6D**	**Kukri_c31995_1948(C**)	**84.37–85.82**	**wsnp_Ra_c13881_21836489**	**−32.98**	**8.5**	**11.9**
	QWPLA.ucl-1B	Tdurum_ contig45965_563(C)	182.03–183.41	cfa2292(C)	−26.03	5.4	7.7
	QWPLA.ucl-5A	BS00065292_51(C)	96.27–96.72	Excalibur_c766_462(C)	25.78	5.2	6.3
	QWPLA.ucl-7B	Tdurum_ contig10932_375(C)	20.19–20.65	GENE.4337_558(C)	−25.37	5.2	7.5
	QWPLA.ucl-4D	wsnp_Ex_ c34252_42593715	11.37–11.54	IAAV5607	−24.01	4.5	5.8
	QWPLA.ucl-3D.3	Kukri_c1764_840(C)	102.61–102.97	wPt.0485	−20.70	3.5	5.1
	QWPLA.ucl-3D.2	TA004904.1360	29.81–38.07	barc0042(C)	20.29	3.2	4.5
	QWPLA.ucl-5D.1	BS00000020_51(C)	0–21.83	Kukri_rep_c115283_502(C)	−23.08	3.1	4.0
	QWPLA.ucl-3B	Jagger_c342_119(C)	22.53–22.99	Excalibur_c27658_264(C)	−18.79	2.9	4.1
	QWPLA.ucl-3A	Ex_c6864_583(C)	75.28–77.19	BobWhite_c52043_344(C)	18.09	2.6	3.8
	QWPLA.ucl-4B	BobWhite_c20051_53(C)	53.33–54.7	barc0340a(C)	−16.76	2.3	3.1
	QWPLA.ucl-6B.2	wsnp_Ex_ c19525_28494827	110.19–110.64	BS00010016_51(C)	−16.13	2.2	2.6
	QWPLA.ucl-1D	BS00065168_51(C)	1.06–6.13	wsnp_JD_c3091_4079762(C)	16.50	2.1	2.9
	QWPLA.ucl-7A.2	GENE.4672_55(C)	118.49–118.95	Excalibur_c1791_819(C)	−15.68	2.1	2.8
	QWPLA.ucl-7A.1	wsnp_Ku_ c4615_8326355(C)	19.04–27.85	BS00106739_51(C)	−16.25	2	2.6
	QWPLA.ucl-2D	RAC875_c39665_175(C)	81.1–85.23	Ex_c2115_3369(C)	15.55	1.9	2.4
	QWPLA.ucl-6B.1	Ex_c20409_854(C)	36.56–40.23	Ku_c2392_1692(C)	15.06	1.9	2.2
	QWPLA.ucl-6A	wsnp_Ku_ c1318_2624758(C)	95.01–115.87	RFL_Contig5722_537(C)	−16.13	1.7	2.4
	QWPLA.ucl-5D.2	wsnp_Ex_c1278_2449191	83.87–132.4	stm0519actcb	−15.24	1.2	1.6
	QWPLA.ucl-3D.1	Tdurum_contig9514_807	13.8–15.87	Excalibur_c878_1249	−11.82	1.1	1.4
	QWPLA.ucl-7D	BS00110124_51(C)	6.44–6.89	gwm0635	−6.32	0.4	0.6
WPSLA	**QWPSLA.ucl-4A**	**D_GCE8AKX01AOOSX_177(C**)	**56.09–56.54**	**wsnp_Ku_c3081_5777347(C**)	**4.12**	**11.1**	**7.08**
	**QWPSLA.ucl-3A.1**	**Kukri_c18529_397**	**19.29–21.12**	**wsnp_Ex_c44375_50444862**	**−4.00**	**10.1**	**5.99**
	**QWPSLA.ucl-5B**	**BobWhite_c8048_663(C**)	**79.57–80.03**	**BobWhite_c45340_368(C**)	**3.85**	**9.8**	**5.03**
	**QWPSLA.ucl-3A.2**	**BS00025191_51(C**)	**67.96–75.28**	**Ex_c6864_583(C**)	**3.98**	**9.6**	**5.53**
	QWPSLA.ucl-6A	wsnp_Ex_c2389_4479352(C)	41.61–81.29	barc0353b(C)	−4.48	8.5	4.57
	QWPSLA.ucl-2B	wsnp_Ra_c54256_57628288(C)	55.24–56.16	Ra_c105116_550(C)	−3.28	7.3	3.27
	QWPSLA.ucl-7A	barc0281(C)	81.31–81.77	BS00065822_51(C)	−3.26	7.1	4.11
	QWPSLA.ucl-3D	wsnp_Ex_rep_ c101732_87042471(C)	27.93–29.81	TA004904.1360	3.15	6.5	3.83
	QWPSLA.ucl-2A	BS00096927_51(C)	135.2–135.66	CAP12_c575_105(C)	−2.49	4.3	2.49
	QWPSLA.ucl-3B	BS00079988_51	7.35–7.81	wPt.2757(C)	−2.35	3.9	2.31
	QWPSLA.ucl-4D	wsnp_Ex_rep_ c67296_65839761(C)	0–0.46	RFL_Contig2797_576(C)	−2.25	3.4	1.96
	QWPSLA.ucl-5A	BS00028356_51(C)	151.77–162.01	BS00022646_51(C)	2.30	3.4	1.95

^a^ See abbreviations for details. ^b^ The QTL name. ^c^ The name of the flanking left marker. ^d^ The position (cM) of the two flanking markers. ^e^ The name of the flanking right marker. ^f^ The effect of RAC875 allele at the considered locus. ^g^ The percentage of genetic variance explained by the QTL. ^h^ Logarithm of odds (LOD) score of the QTL. Highlighted areas in bold reflect QTL that are considered to be robust (%Var higher than 8%, LOD higher than 2.4 and QTL peak defined in a region narrower than 18 cM).

Six QTL were detected for SLP, collectively explaining 83.4% of the genetic variance. Four of these QTL were specific to daytime TR (i.e. also detected for TR1.1 or TR3.7, Table 2). The most robust of these QTL (QSLP.ucl-5A) was the same major 5A QTL that was common for TR1.1 and TR3.7, and explained the largest percentage of the observed genetic variance (25.4%) among all the 68 QTL. Overall, three out of four of these major QTL conferred a decrease in the response of whole-plant TR to VPD through the RAC875 alleles.

A larger number of QTL were identified for TRN, together explaining 71.1% of the genetic variance. The QTL were specific to this trait with the exception of QTRN.ucl-2D, QTRN.ucl-6A.2 and QTRN.ucl-6D, which were also detected for SLP, NTIL and WPLA, respectively. The QTL QTRN.ucl-5A.2 was the most robust of the trait-specific QTL, explaining 11.7% of the total genetic variance (Table 2). Except for one, all these QTL conferred a decrease in TRN as a result of the presence of the allele of the conservative line RAC875.

Five trait-specific QTL were identified for stomata density traits (Table 2). The QTL QSDAD.ucl-7A was the most robust of these, explaining 23.6% of the genetic variance. Surprisingly, none of the QTL detected were common for SDAB and SDAD and for all detected QTL, the RAC875 allele increased both SDAB and SDAD. Five QTL were detected for NTIL, two of which were shared with WPLA and TRN on chromosomes 2D and 6A, respectively. Three potentially major QTL for this trait were detected on chromosomes 2D, 5B and 4D (Table 2).

The largest group of 23 QTL was detected for WPLA, which altogether explained 87.8% of the total genetic variation of the trait. With the exception of three QTL that overlap with SLP, TRN and NTIL QTL, 20 WPLA QTL were trait-specific, the majority of which having relatively small effects (Table 2). Among these, three major QTL were identified, the most robust being the QTL QWPLA.ucl-5B (LOD=18.7), which individually explained 15.8% of the total genetic variance. For the large majority of WPLA QTL including two of the three major ones, the RAC875 allele decreased the whole-plant leaf area (Table 2).

Collectively explaining 85% of the total genetic variance for the trait, 12 QTL were detected for WPSLA, none of which co-localized with QTL for WPLA and most of which were specific to this trait, with the exception of two QTL on chromosomes 5B and 6A that were shared with TR3.7 and NTIL, respectively. Overall, the RAC875 allele conferred a decrease in WPSLA for the majority of the QTL detected.

### Gene identification in the peak region of QSLP.ucl-5A

The peak region of the major locus QSLP.ucl-5A was delineated from 110.99 to 113.75 cM including a total of 53 SNP. After removing SNP corresponding to the same locus (Wang *et al.*, 2014), we found 29 unique HC gene predictions for the interval. Of those, a first group of 13 genes were found in the PopSeq map delineating the corresponding region from 54.88 to 55.66 cM (IWGSC, 2014), which included a total of 56 wheat genes. The second group of 16 genes was only found in the RAC875/Kukri region but absent in PopSeq, making up a total of 72 genes that were mapped in the peak region of QSLP.ucl-5A.

**Fig. 5.**
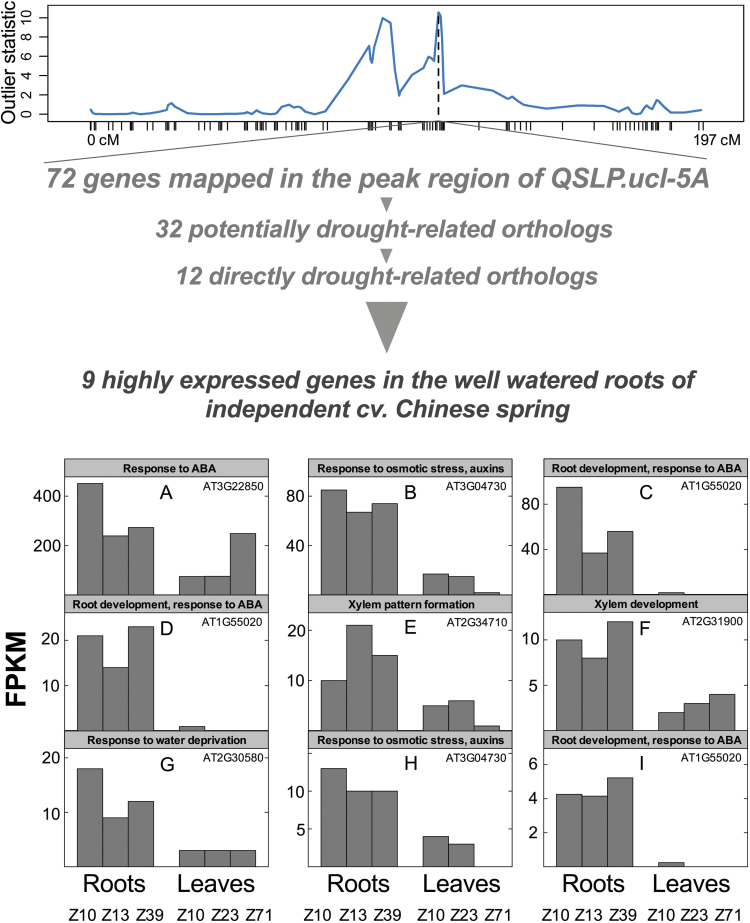
Putative genes in the peak region of the major quantitative trait locus QSLP.ucl-5A controlling whole-plant TR response to increasing evaporative demand. Among the group of 72 genes identified, 12 were found to be potentially directly involved in drought tolerance (see Supplementary Table S1). (A–I) RNA-Seq profiles of nine genes out of the group of 12 that were found to be highly expressed (10–363 FPKM) in the roots of an independent commercial wheat cultivar (cv. Chinese Spring) under well-watered conditions at different phenological stages indicated by Zadoks stages. Loci references of orthologous Arabidopsis genes in the TAIR database are reported in each panel.

Rice and Arabidopsis homologs were found for 68 of these 72 genes and within this group, a search in TAIR indicated that at least 32 homologs are potentially drought-related (Supplementary Table S1 available at *JXB* online). The search identified a first group of 12 closely drought-tolerance related genes. These were directly involved in water transport, response to abscissic acid, osmotic stress, water stress, heat stress, root development response to ABA and plant hydraulics traits (i.e. xylem development). A second group was identified, consisting of another 13 stress-related, but non-drought specific genes. A final group consisted of seven non-stress response genes, but whose functions are likely to be involved in TR response to VPD, such as cell growth and elongation, leaf development, temperature response and photosynthesis (Supplementary Table S1). Interestingly, nine wheat HC gene predictions out of the first group of 12 drought-related homologs were found to be highly and specifically expressed in the roots of the wheat cultivar Chinese Spring grown under well-watered conditions (Fig. 5).

## Discussion

### There is a strong genetic basis for whole-plant transpiration response to increasing VPD in wheat

Since the 1990s, several investigations have shown that transpiration-related traits such as single leaf conductance were heritable and genetically structured in wheat (e.g. Condon *et al.*, 1990; Rebetzke *et al.*, 2003). However the approach of circumventing leaf-to-leaf variation while taking into account the driving effect of VPD in order to phenotype response curves within an entire population was not attempted. In pearl millet, Kholová *et al.* (2012) characterized whole-plant TR in a mapping population, but those measurements were made over five different days at two different VPD levels. On soybean, Sadok and Sinclair (2009) attempted to construct entire TR vs. VPD response curves for recombinant inbred lines resulting from a cross between a ‘conservative’, drought-tolerant line and a check commercial cultivar, but those were limited to only 22 genotypes. Our approach allowed for containing the key limitations (see final section) underlying the precise phenotyping of TR response curves to increasing VPD simultaneously for hundreds of plants.

When compared to the diversity of the responses previously identified in wheat (i.e. Schoppach and Sadok, 2012), the systematically linear nature of the relationship linking TR to increasing VPD appears striking. The large variation in the slopes (Fig. 1) indicates a strong variation in canopy conductance in this population. This suggests that under our experimental conditions, leaf and/or root hydraulic conductance were unlikely to have changed as VPD increased. In this regard, assuming additive effects of the different QTL, our experimental approach appears to have captured the 83.4% of the loci controlling whole-plant leaf conductance in this population while revealing major QTL. One of those (QSLP.ucl-5A) individually explained the highest genetic variance among all detected QTL in this analysis. This QTL was detected on chromosome 5A, and importantly, was specific to daytime TR response to VPD. The above, combined with (i) the particularly narrow region defining the peak of this QTL (0.45 to 0.92 cM), (ii) the associated high heritabilities (0.7 and 0.63, respectively, for traits TR1.1 and TR3.7) and (iii) the fact that the RAC875 allele at this locus confers a conservative TR, made this QTL a good target for a further genetic dissection.

Using the high-resolution map of the QTL plot with 53 SNP markers underneath, QSLP.ucl-5A was anchored onto the chromosome-based draft sequence of the wheat genome and for gene discovery (IWGSC, 2014). The dissection of the peak region of QSLP.ucl-5A revealed 12 genes that were drought-related in Arabidopsis, including DRIP1, a negative regulator of DREB2A, a transcription factor involved in drought tolerance whose expression levels were found to be highly increased in the roots of soybean mutants exposed to water deficit (Engels *et al.*, 2013). Consistently, a large majority (nine) of these genes were preferentially expressed in the well-watered roots of the cultivar Chinese Spring, with annotations suggesting roles in root development and response to ABA, water transport and xylem formation (Supplementary Table S1). Those findings are strongly in line with our previous report where we traced back the conservative behavior of the drought tolerant line RAC875 to a lowered root hydraulic conductivity that was linked to a limited metaxylem size in the seminal roots, together with a restricted trans-membrane water transport – likely involving aquaporins (Schoppach *et al.*, 2014b). Several other potentially drought related genes were involved in leaf-based gas-exchange related processes (e.g. photosynthesis, temperature response, leaf expansion), consistently with the involvement of leaf hydraulics in TR response to VPD. Further research is necessary to confirm these genes and unravel their physiological roles in order to substantiate their relevance at the whole plant level, particularly in terms of whole-plant water use response to evaporative demand.

### Trade-offs between traits involved in transpiration response to evaporative demand

There was a strong, negative genetic and phenotypic correlation between SLP and WPLA, confirming previous long term studies on wheat (Schoppach and Sadok 2013) and soybean (Devi *et al.*, 2015). Such correlation is consistent with the recent investigation of Pantin *et al.* (2013), who reported a negative relationship between plant size and transpiration in Arabidopsis. This correlation was mediated by an ontogeny-dependent acquisition of sensitivity to ABA in the leaves, which progressively limits transpiration as the leaves get older and the plant total leaf area gets larger. Interestingly, this acquisition was triggered by the progressive exposure of bigger leaves to higher evaporative demand, suggesting a developmental link between TR response to VPD and ABA, which is consistent with the nature of the genes identified in the peak region of QSLP.ucl-5A and also with the divergent influence of *Ppd-D1* and *Ppd-B1* on SLP and WPLA (Fig. 3). In any case, if the reported correlations are valid beyond this population, they suggest that there are trade-offs to selecting for low TR under high VPD, which might lead to selecting plants with larger leaf areas. However, the fact that only 1 QTL co-localized for SLP and WPLA seems to indicate possibilities for selecting for TR independently from leaf size.

Despite the significant correlations between WPLA and WPSLA (Table 1), all of the QTL detected for these traits were distinct, indicating that they do not capture the same physiological processes, therefore offering two distinct sources for breeding drought-tolerant germplasm. Indeed, WPLA represents the total evaporative area of the plant, and is consequently a trait whose variation can lead to drought tolerance in environments requiring evaporative surface adjustment to save water, while WPSLA alternatively encapsulates a drought adaptation strategy that involves maximizing the photosynthetic assimilation per unit leaf area and/or increased early vigor (Rebetzke *et al.*, 2004). However, in comparison to transpiration traits, the high number of medium-effect QTL detected for WPLA and WPSLA in this study makes it relatively more challenging to use these QTL in breeding programs. In such efforts, the focus should be clearly on the major QTL identified for these traits, and especially QWPLA.ucl-5B.

### Both abaxial and adaxial stomata densities have a strong genetic basis but they do not correlate well with whole-plant traits

A unique characteristic relative to the stomata densities measured in this study was that robust QTL were detected for both the abaxial (SDAB) and adaxial (SDAD) (Table 2). We did not find previously published QTL data on stomata density for wheat, but our values appear to compare very favorably with the literature dealing with other crops such as rice (e.g. Laza *et al.*, 2010). Further, the stomata density traits were only weakly correlated with the other traits both phenotypically and genetically (Table 1), indicating an independent genetic control. While a strong genetic correlation was observed between SDAD and SDAB (Table 1), it is rather surprising that the QTL for SDAD and SDAB were different. This indicates that the abaxial and adaxial sides are not fully controlled by the same genetic and developmental processes and that they do not necessarily carry the same physiological function. However, on the basis of the low significance levels of the correlations between stomata densities and whole-plant water use traits, it doesn’t seem that SDAD and/or SDAB capture physiological information relevant to improving yield under drought. Further research, for instance involving gas exchange phenotyping of NILs or genotypes segregating for the region of the major SDAD and SDAB QTL, will certainly help resolve the nature of involvement of stomata densities in whole-plant water use.

### Nocturnal transpiration has a genetic basis in wheat

One major finding of this investigation was that TRN was highly heritable and controlled by specific loci of the wheat genome. This finding is the first to confirm the hypothesis of existence of a genetic basis of nocturnal transpiration in plants, first established by Christman *et al.* (2008) who identified one QTL controlling nocturnal stomata conductance in Arabidopsis. Further, the present finding is unique in that whole-plant night-time transpiration – instead of single leaf-based gas exchange – was measured over several hours, thereby limiting confounding effects resulting from instantaneous measurements or from measuring single leaf segments.

Another significant finding was that most of the QTL identified for TRN were specific to this trait (Table 2), indicating that whole-plant nocturnal transpiration is under the control of distinct physiological processes. However, consistent with Christman *et al.* (2008), SLP and TRN were found to be positively correlated both phenotypically and genetically (Table 1), indicating that any selection that operates on daytime TR is likely to influence night-time TR. Given the origin of the genetic material (south Australian drought-adapted parents), this suggests that although limited, TRN might confer some favorable physiological advantage that benefits productivity under drought. In wheat, TRN was found to be non-negligible in south Australian conditions (Rawson and Clarke, 1988) and more recently was shown to be under the control of night-time VPD and speculated to be involved in drought tolerance by Schoppach *et al.* (2014a), but the nature of this involvement remains unclear. By uncovering QTL such as QTRN.ucl-5A.2, the present investigation offers a new way to address this challenge. Investigating the physiological basis of TRN variation among NILs for this QTL will unravel the ecophysiological and agronomic value of this trait.

### A complex and differential involvement of phenology genes in regulating transpiration-related traits

The 143 DH lines that were used in our study were selected from a group that flowered in a narrow window, in order to limit interaction with phenology (e.g. Bonneau *et al.*, 2013). Despite this, the effects of the maturity genes were substantial and different, revealing a much stronger effect of *Ppd-D1* in regulating most of the traits investigated, with the exception of stomata densities. Unexpectedly, the RAC875 alleles for *Ppd-D1* conferred an increase in daytime and night-time transpiration (Fig. 3). However, the same allele also conferred a potentially conservative behavior by strongly decreasing whole-plant leaf area (WPLA), further confirming the hypothesis of a trade-off between these two types of traits, as outlined earlier in the discussion. At any rate, these findings confirm the long suspected possibility that both genes have pleiotropic effects on plant growth and development (Cockram *et al.*, 2007), while suggesting that these effects involve hydraulics and gas exchange response to a major abiotic stress (i.e. VPD). Consistent with Pantin *et al.* (2013), this indicates that at least some of the whole-plant hydraulic and photosynthetic physiological functions are developmentally controlled and therefore are not necessarily expressed identically across the crop cycle. If confirmed, this observation implies that drought tolerance traits evolve with time as the crop grows, which point to the increasing necessity of taking into account such time-dependent effects when modeling or examining the relationship between a putative drought tolerance trait and yield.

### Caveats inherent to phenotyping TR response to increasing VPD

This investigation revealed two main challenges that need to be considered when phenotyping TR response curves to increasing VPD. The first deals with the caution that should be applied in replicating this type of experiment. Prior to this study, efforts to phenotype the entire population three times at the seedling stage under different conditions using a hydroponic system failed to reveal heritable TR and developmental traits. This led to phenotyping the population with the current protocol, i.e. at a more advanced stage, in large custom-made pots. This protocol appeared to be repeatable since the 2015 validation experiment confirmed the TR responses of eight genotypes that captured the variability in SLP of the entire population. However, as indicated by the strong effects of the maturity genes, it should be noted that replicating the experiment at a later/earlier stage is likely to strongly influence the QTL detection. This also applies to situations where experiments are undertaken under significantly different growth conditions, which were reported to influence the TR vs. VPD curves in a genotype-dependent fashion, on maize (Yang *et al.*, 2012) and wheat (Schoppach and Sadok, 2013).

A second challenge deals with the followed approach for imposing the VPD treatments. Unavoidably, the achieved VPD was the result of both variation in T and RH as a function of the time of the day, so that it is impossible to uncouple T, PPFD and VPD effects on TR, similar to other reports (Kholová *et al.*, 2010, 2012). Doing so requires phenotyping the entire population in an environment where T and PPFD stay constant, while dramatically varying RH, which is impossible outside a growth chamber. In our experimental setting, the increase in T over time was concomitant with a decrease in RH, while PPFD stayed above saturation levels for most of the sequence. This makes it reasonable to ascribe TR increases largely to increases in T and RH-driven VPD as the main driving force. In a previous investigation (Schoppach and Sadok 2012), we confirmed the repeatability of this approach over two independent experiments that were carried out each over two successive days in the growth chamber and the greenhouse, despite different light environments where PPFD stayed above 400 μmol m^−2^ s^−1^. Considering the logistical impossibility of phenotyping the entire population inside a growth chamber and the statistical necessity of characterizing simultaneously all individuals, future phenotyping efforts – especially those under naturally fluctuating conditions – will have to take into account the above considerations.

## Supplementary data

Supplementary data are available at *JXB* online.

Table S1. List of genes identified in the peak region of the major QTL on chromosome 5A (QSLP.ucl.5A) controlling whole-plant TR response to increasing vapor pressure deficit (VPD).

Supplementary Data

## Abbreviations:

cM: centiMorgan
CSS: chromosome survey sequences
DArT: diversity arrays technology
DH: double haploid
FPKM: fragments per kilobase of exon per million fragments mapped
HC: high confidence
IWGSC: International Wheat Genome Sequencing Consortium
LOD: logarithm of the odds
NIL: near isogenic line
NTIL: number of tillers
QTL: quantitative trait loci
RH: relative humidity (%)
RNA-Seq: RNA sequencing
SDAB: abaxial stomata density (mm^−2^)
SDAD: adaxial stomata density (mm^−2^)
SLP: slope of the linear regression linking daytime TR and VPD (mg H_2_O m^−2^ s^−1^ kPa^−1^)
SNP: single nucleotide polymorphism
SSR: simple sequence repeat
T: air temperature (°C)
TR: transpiration rate (mg H_2_O m^−2^ s^−1^)
TR1.1: TR at a VPD of 1.1 kPa
TR3.7: TR at a VPD of 3.7 kPa
TRN: night-time TR
TSW: tiller and spike dry weights
VPD: vapor pressure deficit (kPa)
WGAIM: whole genome average interval mapping
WPLA: whole-plant leaf area (cm^2^)
WPSLA: whole-plant specific leaf area (cm^2^ g^−1^)
ZS: Zadoks growth scale.
