# Supplementary material for: High resolution mapping of traits related to whole-plant transpiration under increasing evaporative demand in wheat
Source: J Exp Bot. 2016 Mar 20;67(9):2847–60. doi: 10.1093/jxb/erw125 (PMC4861027; doi:10.1093/jxb/erw125)
Supplement: Supplementary Data [file supp_67_9_2847__index.html]

High resolution mapping of traits related to whole-plant transpiration under increasing evaporative demand in wheat — High resolution mapping of traits related to whole-plant transpiration under increasing evaporative demand in wheat — Supplementary Data 

# High resolution mapping of traits related to whole-plant transpiration under increasing evaporative demand in wheat

## Supplementary Data

Data files

- supplementary\_table\_S1.xlsx - Supplementary Data
